# Systematic Literature Review to Determine Existing Data on the Growth of *Listeria monocytogenes* in Ready-to-Eat Foods Performed Based on the European Union Reference Laboratory (EURL) Lm Technical Guidance Documents

**DOI:** 10.3390/foods15081402

**Published:** 2026-04-17

**Authors:** Andrea Singer, Roger Stephan

**Affiliations:** Institute for Food Safety and Hygiene, University of Zurich, Winterthurerstrasse 272, CH-8057 Zurich, Switzerland; stephanr@fsafety.uzh.ch

**Keywords:** *Listeria monocytogenes*, challenge test, growth potential, maximum growth rate, ready-to-eat food, RTE, food safety

## Abstract

With rising incidence in recent years, Listeriosis, a severe foodborne disease in humans primarily transmitted through ready-to-eat (RTE) foods contaminated with *Listeria monocytogenes*, became the most severe zoonotic disease in the European Union (EU) in 2024 with the highest hospitalization and mortality rates, prompting stricter regulatory requirements under Regulation (EC) No 2073/2005 and its recent amendments. This systematic literature review aimed to evaluate the availability, validity and quality of published challenge test data on the growth potential and maximum growth rate of *Listeria monocytogenes* in RTE foods to identify data gaps and, if possible, to support the derivation of a classification of RTE foods into the two existing regulatory categories, a and b (not able and able to support the growth of *Listeria monocytogenes*). Conducted in accordance with the Preferred Reporting Items for Systematic Reviews and Meta-Analyses (PRISMA) guidelines and the Cochrane Handbook, a comprehensive database search was done to identify eligible challenge test studies on *Listeria monocytogenes* growth in RTE foods, followed by structured screening and quality assessment based on the EURL Lm Technical Guidance Documents. A limited and heterogeneous body of published challenge test data on the growth potential and maximum growth rate of *Listeria monocytogenes* in RTE foods was identified, with substantial data gaps across multiple food groups, precluding meta-analysis and limiting regulatory applicability under the current regulations. Overall, the available literature is insufficient to reliably support regulatory classification or to enable direct extrapolation by food business operators (FBO), underscoring the need for product-specific investigations and food group-specific guidance for food safety.

## 1. Introduction

Listeriosis is a foodborne disease in humans caused by *Listeria monocytogenes*, a pathogenic gram-positive, rod-shaped bacterium belonging to the genus *Listeria* [1]. *Listeria monocytogenes* is non-spore-forming, aerobic and facultatively anaerobic, and genetically diverse with four evolutionary lineages (I–IV), 13 serotypes and four major molecular serogroups. While historically, serotype 4b was the most prevalent serotype in clinical Listeriosis cases, serotype 1/2a has more frequently been linked to human Listeriosis in the last decade. *Listeria monocytogenes* is psychotrophic, able to grow at refrigeration temperatures, and its occurrence is ubiquitous.

Almost all Listeriosis cases (99%) are transmitted through food. Contamination of food with *Listeria monocytogenes* can take place at any step of the harvesting, processing, preparation, packaging, transportation or storage of food. RTE foods pose a particular risk in the transmission of *Listeria monocytogenes* to humans as they are food products intended for direct human consumption “without the need for cooking or other processing effective to eliminate or reduce to an acceptable level the micro-organism of concern” [1] (p. 40).

There are several types of Listeriosis. The maternal–neonatal form can lead to abortions and neonatal infections. Bacteremic forms can lead to septicemia. The neuro-meningeal form can cause meningitis and meningoencephalitis. Finally, the gastroenteric form leads to fever, nausea, vomiting and diarrhea. An incubation time of up to 67 days, depending on the Listeriosis type, can make it difficult to detect as the cause of illness. Young, old, pregnant and immunodeficient people (YOPI group) are at particularly high risk for more severe forms of the disease.

Listeriosis cases in humans have been rising in recent years, reaching 3041 reported cases in the EU in 2024—the highest since 2007 [2]. Although relatively rare compared to other foodborne infections, it is the most severe zoonotic disease, with a hospitalization rate of 97.3% among the 3041 reported cases with an available status. It also caused the highest number of deaths (*n* = 301, 15.6% of clinical cases), exceeding salmonellosis (*n* = 116) and campylobacteriosis (*n* = 76).

Current regulations on microbiological criteria for foodstuffs in the EU state that FBOs must ensure that their products contain less than 100 colony forming units (cfu) per gram (g) (cfu/g) throughout their shelf-life, with shelf-life being defined as “either the period corresponding to the period preceding the ‘use by’ or the minimum durability date” [3] (L 338/5). To ensure this, the regulation distinguishes between RTE foods that are not able to support the growth of *Listeria monocytogenes* (group a) and those that are able to support its growth (group b). RTE foods are automatically classified as not supporting the growth of *Listeria monocytogenes* (group a) when specific intrinsic factors are met, including a pH ≤ 4.4, a water activity (aw) ≤ 0.92, a combination of pH ≤ 5.0 and aw ≤ 0.94, or a shelf-life of less than five days. Additional RTE foods may also be assigned to group a, provided that sufficient scientific justification is available. For RTE foods in group a, a food safety criterion of less than 100 cfu/g in five food samples applies. In contrast, RTE foods that support the growth of *Listeria monocytogenes* (group b) must demonstrate the absence of the pathogen in five samples of 25 g each. This demonstration may be achieved through challenge tests assessing the growth potential or maximum growth rate or through durability studies, in accordance with the EURL Lm Technical Guidance Documents [1,4,5,6,7].

A recent amendment of the EC regulation No 2073/2005, entering into force on 1 July 2026, extends the previous regulation for group b to all stages where the product is placed on the market [8]. Consequently, the amendment extends regulatory obligations to retailers and further businesses placing the products on the market; however, their responsibility is limited to maintaining proper storage conditions (e.g., refrigeration), whereas primary responsibility for ensuring compliance remains with the manufacturing FBO.

The reason for the recent changes to stricter regulations were the observations made by the European Food Safety Authority (EFSA) on Listeriosis, stating that the number of deaths from foodborne outbreaks and cases by *Listeria monocytogenes* was one of the highest numbers reported in the last 10 years and that it therefore is crucial that food safety criteria for *Listeria monocytogenes* can offer a high and consistent level of protection of consumers throughout the food chain [9]. In Switzerland, the stricter regulation of the EU has been adopted and the applicable regulation was adapted in summer 2025 [10].

Although the current regulatory changes in the EU and in Switzerland strengthen consumer protection by requiring compliance with the <100 cfu/g throughout their entire shelf-life and not only until placed on the market, the classification of a RTE food into group a or b remains challenging. Therefore, the aim of this systematic literature review was to gather published data on the growth of *Listeria monocytogenes* in various RTE foods. The review focused on the assessment of the amount, validity and quality of published challenge test data assessing the growth potential and maximum growth rate of *Listeria monocytogenes* in RTE foods as defined in the EURL Lm Technical Guidance Documents [1,4,5,6,7]. Furthermore, the goal was to identify gaps in the existing literature data and to derive further guidance to support the classification of the RTE foods in the two regulatory groups.

## 2. Materials and Methods

This review followed the PRISMA guidelines [11]. Additionally, the Cochrane handbook was used [12].

The full study protocol can be assessed on the International Prospective Register of Systematic Reviews (PROSPERO, identification code: CRD420251151869) [13].

A search strategy was set up using multiple electronic databases. As a search framework, the universal “Patient, Intervention, Control, Outcome” (PICO) method, as described in the Cochrane handbook, was used to develop a search strategy in which keywords were combined [12]. A systematic search of the published literature was carried out using PubMed and Scopus. Additional searches were done on Multidisciplinary Digital Publishing Institute (MDPI), EFSA publications, Bundesamt fuer Lebensmittelsicherheit und Veterinaerwesen (BLV), Technical University of Denmark (DTU) research database and the Risikobewertungsinstitution in Deutschland (BfR). For gray literature, supplementary keyword generation was supported by ChatGPT (Model 5-2, free version), and all identified records were subsequently retrieved and verified through the respective primary sources. Moreover, a search on the Prospero Network was carried out to preclude that another systematic review has covered the same objective. No other method of identifying studies was used. The electronic search strategy was reviewed by the co-examiner using the Peer Review of Electronic Search Strategies (PRESS) checklist [14]. No filters or limitations and no search date restrictions were used. The searches were carried out up until 31 May 2025. The PICO framework, the search terms and the complete search strategy can be found on the PROSPERO website (identification code: CRD420251151869) [13].

Eligibility criteria were created following the PICO framework as described in the Cochrane handbook and served as a foundation for creating the inclusion and exclusion criteria [12]. An article was included if its full text was available in English or German and if it was publicly available or could be ordered without additional costs through Universitaere Zentralbibliothek (UZB) Zürich or Eidgenoessische Technische Hochschule (ETH) Zürich. Articles had to be studies on RTE food and had to include challenge tests to assess the growth potential or maximum growth rate of *Listeria monocytogenes,* both as defined in the EURL Lm Technical Guidance Documents [1,4,5,6,7]. Studies that deviated from this Guidance Document, reviews, guidelines or data collected otherwise were excluded.

**Figure 1:**
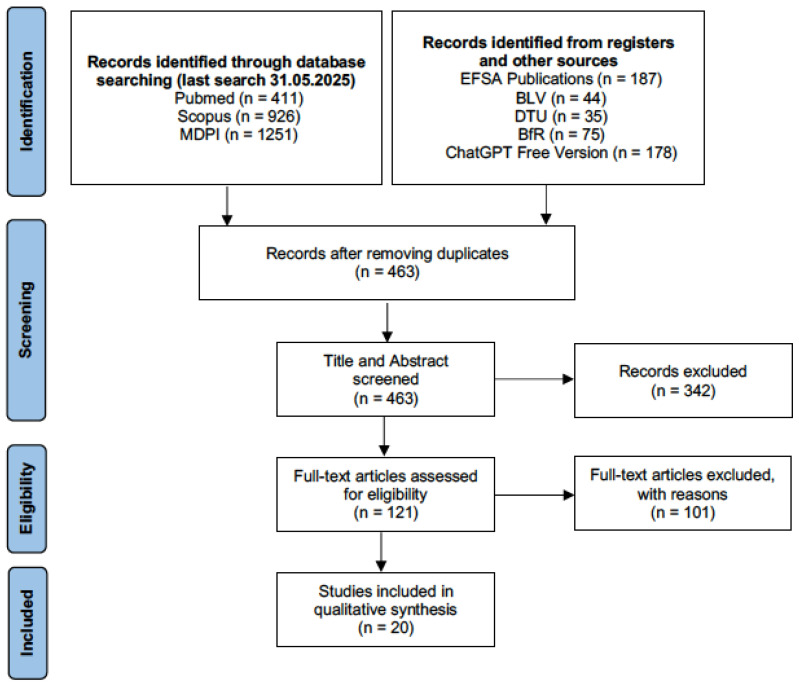
Adapted PRISMA flow diagram.

The search results obtained were exported to Zotero (Version 7.0.30) and further transferred to Microsoft Excel (Version 16.16.27). A two-step approach, as suggested in the Cochrane handbook, was used for study screening [12]. First, only the study title and abstract were considered for study selection. Potentially included studies were further assessed through full-text analysis. The decision on which studies were included in this review (both for title/abstract and full text analysis) was taken according to the eligibility criteria. For every excluded study, the reason for exclusion was recorded in Microsoft Excel (Version 2602). This procedure was done by a single individual. However, doubtful cases and the included studies were all discussed with the co-examiner. The study selection process was outlined in detail in the PRISMA flow diagram, as shown in Figure 1.

The following data extraction was performed in Microsoft Excel using the full text of the included studies. If possible, different RTE food subgroups and batches were separated. Missing data were not included in the calculations but were described as outlined in the Section 3.

The quality of each article was assessed based on compliance with the referenced version of the EURL Lm Technical Guidance Document [1,4,5,6,7]. Studies were classified as compliant, minor or major deviation based on an overall study-level assessment supported by a description of identified deviations. Minor deviations were defined as departures from the guidance document that are unlikely to substantially affect the validity or interpretability of the study results (such as inoculation concentrations close to target contamination levels or missing negative controls). Major deviations were defined as methodological shortcomings with the potential to significantly influence study outcomes or their interpretation (such as deviations in the experimental procedure, such as inoculation during processing instead of after production, or insufficient reporting of key parameters such as storage temperature). In addition, the overall rating also considered the cumulative extent of deviations within a study, with multiple or more substantial deviations leading to classification as a major deviation. To enable quantification, these categories were converted into a numerical quality score.

The validity of the data was evaluated across the domains of methodology, transparency and reporting quality, consistency, representativeness, precision, completeness of evidence, and regulatory applicability, as detailed in the assessment focus of each domain. Judgements of validity (low/moderate/high) reflect the strength of evidence supporting each domain, with low indicating weak support, moderate meaning partial support, and high meaning strong support. For transparency and comparability, judgements were additionally assigned an ordinal score (low = 1, moderate = 2, high = 3), providing an overall validity assessment of the data included in this review.

The descriptive statistics were performed using the built-in Microsoft Excel tools. It is important to point out that no article was excluded during analysis due to its results. However, some studies could not be included in the calculation due to the lack of specific data or due to mixed subgroups.

## 3. Results

### 3.1. Study Selection

The study selection process can be followed in detail in the PRISMA flow diagram in Figure 1. A total of 3107 records were identified through the search strategy in multiple electronic databases, from which 2644 duplicates were removed. Consequently, the title and abstract of 463 remaining records were screened. After further exclusion of 342 records, the full text of 121 remaining articles was analyzed. Of the 121 articles, 66 did not proceed in accordance with the version of the EURL Lm Technical Guidance Document that was applicable at the time of publication and were therefore excluded. An additional 22 articles did challenge tests other than those on growth potential or on maximum growth rate and were consequently excluded. Furthermore, 13 articles were excluded because they were not on RTE food (*n* = 4), *Listeria innocua* was used as a substitute for *Listeria monocytogenes* (*n* = 3), the article was not publicly accessible or only with additional costs (*n* = 3), the *Listeria* species was not mentioned (*n* = 1) or the article was not in English or German (*n* = 1). Thus, the data of 20 studies were included in this systematic review.

### 3.2. Regulatory Framework and Quality Assessment of the Selected Studies

Of 20 included studies, three studies (15.0%) proceeded in accordance with version 2 of the EURL Lm Technical Guidance Document of 2008 [4,15,16,17], nine studies (45.0%) proceeded in accordance with version 3 of the EURL Lm Technical Guidance Document of 2014 [5,18,19,20,21,22,23,24,25,26], one study (5.0%) proceeded in accordance with version 3, Amendment 1 of the EURL Lm Technical Guidance Document of 2019 [6,27], and seven studies (35.0%) proceeded in accordance with version 4 of the EURL Lm Technical Guidance Document of 2021 [1,28,29,30,31,32,33,34]. No study proceeded in accordance with the most current revision of the “Guidance Document on *Listeria monocytogenes* monitoring and shelf-life studies for ready-to-eat foods under Commission Regulation (EC) No 2073/2005 of 18 December 2025 on microbiological criteria for foodstuffs” [7].

Most studies (19/20, 95.0%) included data on challenge tests evaluating the growth potential, whereas four studies (4/20, 20.0%) included data on challenge tests assessing the maximum growth rate.

To evaluate quality, each included study was reviewed for compliance with the referenced version of the EURL Lm Technical Guidance document. A full summary is presented in Table 1.

### 3.3. Validity Assessment of the Included Data

To evaluate the validity of the included studies, the data was reviewed for methodology, transparency and reporting quality, consistency, representativeness, precision, completeness of evidence, and regulatory applicability, as detailed in Table 2.

### 3.4. Categorization into Comparable Food Groups

The available data from the included literature was divided into categories and subcategories in order to form groups of data that are homogeneous enough to be comparable. First, the data of the 20 included studies was divided into the category “EURL Lm Technical Guidance Document Version” to which the individual studies referred. This division was necessary as successive versions of the EURL Lm Technical Guidance Documents differ substantially from one another.

Version 2 (2008) constituted the first comprehensive laboratory guidance under Regulation (EC) No 2073/2005 [3,4], providing detailed procedures for challenge tests and durability studies and establishing baseline methodologies for compliance with microbiological criteria for *Listeria monocytogenes* in RTE foods. Version 3 (2014) fully replaced Version 2, standardizing approaches across EU laboratories and incorporating updated technical protocols, as well as expanded practical guidance on study design and interpretation [5]. Amendment 1 to Version 3 (2019) primarily revised storage and temperature conditions applied in retail-level challenge tests, particularly cold chain profiles, to better reflect observed European practices [6]. Version 4 (2021) represented a major revision, aligning the document with EN ISO 20976-1:2019 on challenge test methodologies for growth potential, lag time, and growth rate determination [1,35]. In December 2025, a new EURL Lm Guidance Document on *Listeria monocytogenes* shelf-life studies was published [7]. However, none of the included studies referenced this version.

For analysis, the included study data were further categorized by “challenge test type”, “food matrix” and “comparable food group”. This led to a total of 29 food groups, of which data were comparable in terms of the version of the EURL Lm Technical Guidance documents followed, the type of challenge test performed, the similarity of the food matrix and in the manufacturing process, the content of the food groups and regarding their intrinsic properties. The data from 28 food groups (28/29, 96.6%) stemmed from one single study each, while the data from one food group (1/29, 3.4%) stemmed from three studies. An overview of all included studies categorized by guideline version, challenge test type, food matrix and comparable food groups can be followed in detail in the evidence map in Table 3.

### 3.5. Challenge Test Conditions Across Selected Studies

A summary of the available data can be found in Table A1 in Appendix A.1.

#### 3.5.1. Number of Batches

Across the 29 food groups, challenge tests were conducted using products originating from between one and 12 production batches. The majority of food groups (*n* = 16, 55.2%) included products derived from three distinct batches. Four food groups (13.8%) were based on products from six different batches, while another three food groups (10.3%) comprised products from two batches. Three food groups (10.3%) relied on products originating from nine batches; for one of these food groups, the nine batches were obtained across three independent studies (contributing three, two and four batches, respectively). One food group (3.4%) included products from four batches, whereas another food group (*n* = 1, 3.4%) was based on a single batch. One further food group (3.4%) incorporated products from 12 distinct batches. The content per batch differed across datasets.

#### 3.5.2. Negative Controls

In total, 15 of 20 studies (75.0%) included negative controls in their challenge tests. As a result, 21 food groups (72.4%) were assessed with a negative control, while seven food groups (24.1%) were assessed without one. For the remaining food group (*n* = 1, 3.4%), where data was derived from three separate studies, two of these studies did not include a negative control, while one study did include a negative control. Among the 21 food groups assessed with a negative control, 15 (71.4%) reported a negative control, or *Listeria monocytogenes* was either absent or not detected in 25 g. For six food groups (28.6%), the outcome of the negative control was not reported. For the food group including data from three different studies, the single study that included a negative control did not report its result.

#### 3.5.3. Choice of *Listeria monocytogenes* Strains

The *Listeria monocytogenes* strains used in the challenge tests varied across all 20 studies included in this review, and none of the studies employed identical strain sets. Both reference strains and wild-type/field strains were used, either in combination (18/29, 62.1%), exclusively as reference strains (5/29, 17.2%), or exclusively as wild-type/field strains (6/29, 20.7%). Most food groups (25/29, 86.2%) were inoculated with a mixture of three strains. In contrast, two food groups (6.9%) were inoculated with a single strain, and one food group (3.4%) was inoculated with two strains. For the food group (*n* = 1, 3.4%) comprising data from three different studies, all studies used a mixture of three strains for inoculation.

#### 3.5.4. Inoculation Concentration and Technique

Inoculation concentrations across the 20 included studies ranged from 10 to 150 cfu/g. Three studies reporting a low inoculation concentration of 10 cfu/g, which is below the levels recommended by the EURL Lm Technical Guidance Documents [1,4,5,6,7], actually employed concentrations ranging from approximately 10 to 100 cf/g, while two studies reporting a low concentration of 32 cfu/g used actual concentrations ranging from 32 to 100 cfu/g. Throughout the 29 food groups, the majority (19, 65.5%) were inoculated with 100 or approximately 100 cfu/g, including the food group comprising data from three different studies, five (17.2%) were inoculated with approximately 10–100 cfu/g, two (6.9%) each were inoculated with 150 cfu/g and 32–100 cfu/g, and one food group (3.4%) was inoculated with 50 cfu/g.

Across the 20 included studies, the applied inoculation techniques comprised surface inoculation in eight studies (40.0%), inoculation in depth in five studies (25.0%), inoculation through a septum in three studies (15.0%), combined surface and in-depth inoculation in one study (5.0%), and contamination during the production process in two studies (10.0%). One study (5.0%) did not report the inoculation technique used. At the food group level, most food groups were inoculated at the surface (13/29, 44.8%), including the food group comprising data from three different studies. This is followed by inoculation in depth (*n* = 7, 24.1%). In four food groups (13.8%), inoculation through a septum was applied, while one food group (3.4%) was inoculated using a combination of surface and in-depth techniques. Three food groups (10.3%) were contaminated during the food production process, and for one food group (3.4%), the inoculation technique was not reported.

#### 3.5.5. Analysis Times

Analysis times varied among food groups, ranging from 0 to 225 days, and were determined by the shelf-life of the RTE foods evaluated as well as by the experimental design of the challenge tests.

### 3.6. Storage Temperatures and Intrinsic Factors Across Selected Studies

A summary of the available data can be found in Table A2 in Appendix A.2.

#### 3.6.1. Storage Temperatures

Storage temperatures varied among food groups and were determined by the type of RTE food and the experimental design of the challenge tests, with temperatures ranging from 2 °C to 37 °C across the tested products. In some challenge tests, a constant storage temperature was maintained throughout the test, whereas in others, temperature conditions varied over time. Several studies comprised multiple challenge tests conducted under different storage temperature conditions. Additionally, certain challenge tests were designed to simulate actual storage conditions proposed by the FBO, while others incorporated abusive storage temperatures to assess product behavior under conditions that may influence food safety.

#### 3.6.2. Intrinsic Factors

The majority of the included studies (16/20, 80%) reported aw values at both the beginning and end of shelf-life. In three studies (15.0%), data on aw values were not reported at either time point, and in one study (5%), initial aw values were missing. At the food group level, aw values at both the beginning and end of shelf-life were available for 23 of 29 food groups (79.3%). For three food groups (10.3%), no aw data were reported, and for a further three food groups (10.3%), data on initial aw values were unavailable.

Data on pH at both the beginning and end of shelf-life were reported for 17 of the 20 included studies (85.0%). In two studies (10.0%), pH values were not provided at either time point, and in one study (5.0%), baseline pH data were unavailable. Consequently, at the food-group level, data on both the beginning and end of shelf-life were reported in 23 of 29 food groups (79.3%). For three food groups (10.3%), pH data were missing at both time points, while for a further three food groups (10.3%), initial pH values were not reported.

Data on aw and pH exhibited substantial variability across the different food groups, reflecting differences in intrinsic physicochemical properties associated with specific food types.

### 3.7. Challenge Test Results

A summary of the available data can be found in Table A3 in Appendix A.3.

#### 3.7.1. *Listeria monocytogenes* Growth

From the 20 included studies, 17 (85.0%) reported data on initial *Listeria monocytogenes* colony counts and 18 (90.0%) provided data on *Listeria monocytogenes* counts at the end of shelf-life. Consequently, at the food group level, for four food groups (4/29, 13.8%), either initial or end-of-shelf-life *Listeria monocytogenes* colony counts were reported. In addition, for one food group (3.4%), data on initial *Listeria monocytogenes* levels were not available.

Both *Listeria monocytogenes* growth and reporting of colony counts differed substantially across the investigated food groups.

#### 3.7.2. Growth Potential

Nineteen studies (19/20, 95.0%) covering 24 food groups (24/29, 82.8%) included data on challenge tests assessing the growth potential. Of those, one study (1/19, 5.3%), including one food group (1/24, 4.2%), did not calculate the growth potential “due to the downward trend in *Lm*” [34] (p. 11).

The reporting of results varied considerably in both format and level of detail. This included the aggregation of batch-specific results into a single summarized value, the presentation of the growth potential exclusively in categorical format (e.g., growth potential < 0.5 log10 cfu/g or >0.5 log10 cfu/g) and, in contrast, the reporting of detailed data for each individual challenge test and batch. Additionally, the reporting structure of challenge test results assessing the growth potential of *Listeria monocytogenes* was inconsistent. Some results were presented at the batch level, whereas others were stratified according to specific experimental conditions, such as ripening time, storage temperature, number of replicates, food type or inoculation technique.

The EURL Lm Technical Guidance Documents classify RTE foods as “able to support the growth of *Listeria monocytogenes*” [1,4,5,6,7], when the growth potential (δ) > 0.5 log10 cfu/g and “unable to support the growth” when the growth potential (δ) < 0.5 log10 cfu/g in the frame of the current regulations on microbiological criteria for foodstuffs in the European Union [3]. From a total of 114 challenge test results across all studies, the growth potential was <0.5 log10 cfu/g for 49 (43.0%); the tested RTE food under the specific challenge test conditions was therefore not able to support the growth of *Listeria monocytogenes*. In contrast, 58 (50.9%) challenge test results had a growth potential of >0.5 log10 cfu/g and were therefore categorized as able to support the growth of *Listeria monocytogenes*. For six challenge test results (5.3%), the growth potential was reported as >0.5 log10 cfu/g in at least one replicate, and in one challenge test (0.9%), the result was reported as >−1.28 log10 cfu/g. A detailed overview of the available growth potential data can be found in Table A3 in Appendix A.3.

#### 3.7.3. Maximum Growth Rate

Four studies (4/20, 20.0%) encompassing data on five food groups (5/29, 17.2%) reported data from challenge tests assessing the maximum growth rate. Among these, two food groups (2/5, 40.0%) comprised RTE fish products. Although following the same EURL Lm Technical Guidance Document version, the data were not comparable due to differences in product characteristics, with one group consisting of a marinated RTE product (marinated salmon tartare), whereas the other group included non-marinated products (tuna fillet, cubed salmon). Similarly, two food groups (2/5, 40.0%) of RTE meat products (pork bars, soft spreadable salami) were not comparable owing to variations in meat type and production processes and following different EURL Lm Technical Guidance Document versions. The remaining food group (1/5, 20.0%) included data on comparable vegetable products (iceberg lettuce, spinach, rocket) regarding their physicochemical properties.

Results on the challenge tests assessing maximum growth rates varied depending on the specific challenge test conditions and on the intrinsic factors of the tested RTE food.

## 4. Discussion

The objectives of this systematic literature review were to assess the amount, validity and quality of published challenge test data assessing growth potential and maximum growth rate of *Listeria monocytogenes* in RTE foods as defined in the EURL Lm Technical Guidance Documents [1,4,5,6,7]. Additionally, the goal was to identify gaps in the existing literature data and to derive, if possible, further guidance to support the classification of the RTE foods in the two regulatory groups.

The analysis identified a limited number of published challenge test studies. Across 20 included studies, data were available for 29 food groups. However, the quantity of data per individual food group was generally low.

In addition to the limited data available, substantial heterogeneity in data reporting was observed. Reported *Listeria monocytogenes* colony counts and growth outcomes varied considerably across studies and were presented in multiple formats, including categorical classifications relative to predefined thresholds (i.e., above or below a specified limit), summary statistics such as means with corresponding standard deviations, qualitative descriptions of pathogen behavior throughout the shelf-life period, and, in some cases, exact numerical counts. Furthermore, inconsistencies were observed in the level of data aggregation. While certain studies reported growth separately for individual batches, others provided only aggregated or summarized results across batches. The overall reporting of challenge test results assessing the growth potential and maximum growth rate of *Listeria monocytogenes* in RTE foods differed considerably in both format and level of detail.

Moreover, experimental conditions varied in terms of the number of batches, *Listeria monocytogenes* strains used, inoculation techniques, inoculation levels, and sampling time points. Such methodological variability further limits the direct comparability of results.

In general, the growth of *Listeria monocytogenes* during the shelf-life of RTE foods is influenced by multiple interacting factors, including intrinsic properties of the food matrix (pH, aw), methodological aspects of the challenge test (inoculation level, inoculation technique, strain selection), and storage conditions (particularly temperature and duration), which play a critical role in determining the extent of *L. monocytogenes* proliferation. While EURL Lm guidance documents define specific thermal abuse conditions, deviations from these standardized scenarios, including FBO-specific temperature profiles, may contribute to variability and limit comparability across studies. Overall, variability across these intrinsic, methodological, and storage-related factors contributes substantially to differences in reported growth outcomes across studies.

The quality of the included data was evaluated based on compliance of the individual studies with the referenced EURL Lm Technical Guidance Document version, focusing on methodological alignment with the recommended procedures and reporting standards outlined in the applicable guidance. While this approach represents a minor deviation from the original protocol, which proposed the Joanna Briggs Institute methodology, it was deemed more appropriate given the regulatory context [36]. Overall, the studies included in this systematic literature review were considered of good methodological quality, demonstrating general alignment with established technical guidance and recommended experimental standards.

The validity of the included data was evaluated based on methodology, transparency and quality of reporting, consistency, representativeness, precision, completeness of the evidence base, and regulatory applicability. This represents a minor deviation from the original protocol, which proposed the GRADE framework [12,37]. However, upon detailed examination, GRADE was considered unsuitable for these experimental challenge test studies due to heterogeneous designs and reporting formats, as it is primarily intended for clinical intervention studies. Consequently, an alternative structured assessment tailored to the methodological and regulatory context of challenge testing studies was applied.

While the majority of the assessed domains were rated as having moderate to high validity, the domains of completeness of evidence and regulatory applicability were judged to be low. Consequently, the overall body of evidence was considered to be of moderate validity. The reduced ratings in these domains were primarily attributable to substantial data gaps across multiple RTE food groups and the heterogeneity of data reporting.

As always, the results of a systematic literature review must be interpreted with caution because of several methodological limitations. First, an inherent risk of selection bias arose from the fact that study selection was conducted by a single reviewer. To mitigate this limitation, all ambiguous cases and all studies ultimately included in the review were discussed with the co-examiner to achieve consensus, so that final inclusion decisions were made jointly by two reviewers.

Although the search included English and German publications, relevant data in other languages may have been missed, introducing potential language bias. While most included studies broadly complied with the referenced EURL Lm Technical Guidance Document, the overall methodological quality varied considerably. To address this heterogeneity, each study was individually assessed for compliance to provide a structured evaluation of quality across studies.

Furthermore, substantial variability in data reporting limited comparability across food groups. The overall scarcity of available data precluded formal meta-analysis or pooled analysis. Instead, a structured validity assessment was applied to critically appraise the evidence.

All these factors negatively impact the overall level of evidence of this systematic review. In particular, the limited amount of available data and the heterogeneity in data reporting across different food groups substantially restrict the robustness and comparability of the findings. Moreover, for several food groups, no relevant literature data was available at all (see Table 3 for an overview of food categories for which literature data are available). This pronounced lack of data underscores that the currently published literature data is insufficient to reliably support the categorization into categories (a) and (b) in accordance with the current regulations on microbiological criteria for foodstuffs in the European Union [3].

Heterogeneity in future studies may be reduced by the most recent revision of the “Guidance Document on *Listeria monocytogenes* monitoring and shelf-life studies for ready-to-eat foods under Commission Regulation (EC) No 2073/2005 of 18 December 2025 on microbiological criteria for foodstuffs” published in December 2025, which provides harmonized, practical support for FBOs and competent authorities [7]. However, as this guidance was published after the literature search cut-off of 31 May 2025, it is not reflected in the studies included in this review. In addition, given its recent publication, only a limited number of studies are likely to have been conducted under these updated recommendations.

## 5. Conclusions

With regard to the safety assessment of the growth of *Listeria monocytogenes* in RTE foods, the currently available literature is insufficient. The existing published data do not provide an adequate basis for FBOs to directly extrapolate findings to their specific products. Consequently, FBOs are required to conduct product-specific investigations on their own initiative, in accordance with the applicable regulatory and methodological framework conditions.

In this context, food sector associations bear responsibility for developing food group-specific guidance that can be applied by FBOs to the foods they produce. Such guidance is essential to support FBOs in ensuring the safety of their RTE products with respect to the potential growth of *Listeria monocytogenes*.

## Figures and Tables

**Table 1:** Quality assessment of included studies.

Author	*EURL LM Technical Guidance Document Version*	Challenge Test (GP, MGR)	Deviation from EURL Lm Technical Guidance Document Version	Compliance with Referenced EURL Lm Technical Guidance Document Version (C, MiD, MaD)	*Quality Score* (C = 1, MiD = 0.5, MaD = 0)
Dalzini et al., 2014 [15]	2, 2008	GP	Inoculation concentration 32–100 cfu/g instead of target contamination level at 50 cfu/g	MiD	0.5
Dalzini et al., 2014 [16]	2, 2008	GP	Inoculation concentration 32–100 cfu/g instead of target contamination level at 50 cfu/g	MiD	0.5
Leong et al., 2015 [18]	3, 2014	GP	No negative control reported (mandatory)	MiD	0.5
Novelli et al., 2017 [19]	3, 2014	GP	Inoculation technique: inoculation of dough during production process instead of after production No negative control reported (mandatory) Calculation of growth potential as difference between LM concentration at each period of sampling and initial LM concentration instead of growth potential as difference between LM concentration at end and beginning of challenge test and consequent categorization into “unable to support growth” if, during all ripening periods, the growth potential values were ≤0.5 log10 cfu/g Initial and end count of LM not reported Storage temperatures not reported Intrinsic factors (aw and pH) not reported	MaD	0
Hunt et al., 2018 [20]	3, 2014	GP	Strain growth at 8 °C and therefore not incubated alongside the product 2 independent batches per food type analyzed in triplicate (except for goat’s milk cheese, where three independent batches were used) No negative control reported (mandatory) Calculation of growth potential as difference between LM concentration at the end and at the beginning or middle of challenge test instead of growth potential as difference between LM concentration at the end and beginning of challenge test	MaD	0
Ruggeri et al., 2018 [17]	2, 2008	GP	Inoculation concentration approx. 10–100 cfu/g instead of target contamination level at 50 cfu/g Inoculation technique not reported No negative control reported as proposed by guidance document	MiD	0.5
Andritsos et al., 2019 [21]	3, 2014	GP	Initial and end count of LM not reported Intrinsic factor (aw value) not reported	MiD	0.5
Marras et al., 2019 [22]	3, 2014	GP	Inoculation concentration approx. 10–100 cfu/g instead of target contamination level at around 100 cfu/g T0 6 h after inoculation instead of directly after inoculation	MiD	0.5
Branciari et al., 2020 [23]	3, 2014	GP, MGR	Inoculation technique: inoculation of meat batter during production process instead of after production Consequent analytical determinations performed during both the production process and the shelf life	MiD	0.5
Culliney et al., 2020 [27]	3, Am. 1, 2019	GP, MGR	Subculture steps of LM strains not reported	MiD	0.5
Eicher et al., 2020 [24]	3, 2014	GP	Use of LM strains isolated from a salmon processing facility instead of the reference strains because they were deemed directly relevant Sushi salmon products used in this study were frozen by the producer immediately after production and stored frozen instead of products being inoculated within 2 days of their production date, as per the guidelines. The other salmon products were stored at −3 °C after packaging. They were then thawed on the day they were inoculated (t = 0)	MiD	0.5
Collu et al., 2021 [25]	3, 2014	GP	Inoculation concentration approx. 10–100 cfu/g instead of target contamination level at around 100 cfu/g Initial and end count of LM not reported	MiD	0.5
Stella et al., 2021 [26]	3, 2014	GP	No deviation	C	1
Vasileiadi et al., 2022 [28]	4, 2021	GP	No reference strains used as proposed by guidance document	MiD	0.5
Tirloni et al., 2023 [29]	4, 2021	GP	No negative control reported (mandatory) Intrinsic factors (aw and pH) not reported	MiD	0.5
Pniewski et al., 2024 [30]	4, 2021	GP, MGR	No reference strains used as proposed by guidance document Measurement unit of MGR not reported	MiD	0.5
Vasileiadi et al., 2024 [31]	4, 2021	GP	No deviation	C	1
Cipriani et al., 2025 [32]	4, 2021	MGR	No deviation	C	1
Ștefan et al., 2025 [33]	4, 2021	GP	No deviation	C	1
Vasileiadi et al., 2025 [34]	4, 2021	GP	Growth potential not calculated due to the downward trend in LM	MiD	0.5

GP: growth potential; MGR: maximum growth rate; LM: *Listeria monocytogenes*; C: compliant; MiD: minor deviation; MaD: major deviation, Am.: Amendment

**Table 2:** Validity assessment of included data.

Domain	Assessment Focus	Judgement (High = 3/Moderate = 2/Low = 1)	Justification
Methodology	Compliance with EURL LM Technical Guidance Document across studies [1,4,5,6,7]	2–3	Mostly compliant (see Table 1)
Transparency & Reporting Quality	Completeness, clarity and reproducibility of reporting of study design, experimental conditions, analytical methods and outcome metrics to ensure reproducibility and reliable evidence synthesis	2	Mostly well described, clear and reproducible; however, heterogeneous reporting of data
Consistency	Degree of concordance in reported growth outcomes across comparable product categories and storage conditions	1	Consistency generally not assessable due to lack of comparable data in specific food groups, except for a small number of studies investigating similar products (cold smoked salmon)
Representativeness	Coverage of relevant RTE food groups, industrial realism	3	Data on representative and realistic RTE food groups
Precision	Reliability of reported µmax and δ estimates based on replication and variability measures	3	Adequate replication and variability reporting
Completeness of Evidence	Presence of data gaps across product categories	1	Presence of data gaps across RTE food groups
Regulatory Applicability	Direct usability for classification in group a or b in accordance with current regulations on microbiological criteria for foodstuffs in the European Union [3]	1	No direct support for group a/b classification due to lack and heterogeneity of published data
**Overall Validity Judgement**	**Integrated assessment across domains**	**1.9**	**Overall moderately valid data due to non-comparability due to large data gaps and heterogeneity in data reporting**

µmax: maximum growth rate; δ: growth potential.

**Table 3:** Evidence map of included studies categorized into EURL Lm Technical Guidance Document version, challenge test type, food matrix and comparable food group.

Guideline Version	Challenge-Test Type	Food Matrix	Comparable Food Group	n (Studies)	References
*Version 4, 2021*	Growth potential	Meat	Pork bars	1	Pniewski et al., 2024 [30]
			Beef in tuna sauce	1	Tirloni et al., 2023 [29]
			Wiener sausage	1	Ștefan et al., 2025 [33]
		Fish	Raw seabass filet	1	Vasileiadi et al., 2024 [31]
		Cheese	Soft Greek Anthotyros cheese	1	Vasileiadi et al., 2022 [28]
			Greek light semi-hard cheese, Greek full-fat semi-hard cheese	1	Vasileiadi et al., 2025 [34]
	Maximum growth rate	Meat	Pork bars	1	Pniewski et al., 2024 [30]
		Fish	Tuna fillet, cubed salmon	1	Cipriani et al., 2025 [32]
			Marinated salmon tartare	1	Cipriani et al., 2025 [32]
*Version 3, Amend. 1, 2019*	Growth potential	Vegetables	Iceberg lettuce, spinach, rocket	1	Culliney et al., 2020 [27]
	Maximum growth rate	Vegetables	Iceberg lettuce, spinach, rocket	1	Culliney et al., 2020 [27]
*Version 3, 2014*	Growth potential	Meat	Pork liver pâté	1	Hunt et al., 2018 [20]
			Salami	1	Novelli et al., 2017 [19]
			Soft spreadable salami	1	Branciari et al., 2020 [23]
			Veal tartare	1	Stella et al., 2021 [26]
		Fish	Cold-smoked salmon, Norwegian smoked salmon	3	Hunt et al., 2018 [20]; Leong et al., 2015 [18]; Eicher et al., 2020 [24]
			Salmon fillet, sushi salmon	1	Eicher et al., 2020 [24]
		Vegetables	Coleslaw	1	Hunt et al., 2018 [20]
			Ready-to-eat salad (radicchio 25%, endive 50%, chicory 25%)	1	Marras et al., 2019 [22]
		Fruit	Fruit salad (grapes, kiwi, melon, pineapple), pineapple	1	Collu et al., 2021 [25]
			Coconut	1	Collu et al., 2021 [25]
			Melon (piel de sapo), melon (cantaloupe)	1	Collu et al., 2021 [25]
		Cheese	Greek feta cheese	1	Hunt et al., 2018 [20]
			Feta cheese-based sauce	1	Andritsos et al., 2019 [21]
			Raw soft goat milk cheese	1	Hunt et al., 2018 [20]
	Maximum growth rate	Meat	Soft spreadable salami	1	Branciari et al., 2020 [23]
*Version 2, 2008*	Growth potential	Meat	Low-fat salami	1	Dalzini et al., 2014 [16]
			Turkey bresaola	1	Dalzini et al., 2014 [15]
			Salsiccia sarda, myrtle-flavored salsiccia sarda	1	Ruggeri et al., 2018 [17]

Amend.: Amendment.

## Data Availability

No new data were created or analyzed in this study. Data sharing is not applicable to this article.

## Supplementary Materials

The study protocol, the PICO framework, the search terms and the complete search strategy can be downloaded at: https://www.crd.york.ac.uk/prospero/ (accessed on 9 April 2026) (identification code: CRD420251151869).

## Abbreviations

The following abbreviations are used in this manuscript:

EURL	European Union Reference Laboratory
LM/Lm	*Listeria Monocytogenes*
RTE	Ready-to-Eat
YOPI	Young, Old, Pregnant, Immunodeficient
FBO	Food Business Operator
EFSA	European Food Safety Authority
PRISMA	Preferred Reporting Items for Systematic Reviews and Meta-Analysis
PROSPERO	Prospective Register of Systematic Reviews
PICO	Patient, Intervention, Control, Outcome
BLV	Bundesamt fuer Lebensmittelsicherheit und Veterinaerwesen
DTU	Technical University of Denmark
BfR	Risikobewertungsinstitution in Deutschland
PRESS	Peer Review of Electronic Search Strategies
UZB	Universitaere Zentralbibliothek Zuerich
ETH	Eidgenoessische Technische Hochschule Zuerich
GP	Growth Potential
MGR	Maximum Growth Rate
C	Compliant
MiD	Minor Deviation
MaD	Major Deviation
N/A	Not Applicable

## Appendix A

### Appendix A.1

**Table A1:** Summary of available data.

Author	*EURL LM Technical Guidance Document Version*	Challenge Test (GP, MGR)	Food Type	No of Batches	Content per Batch	Neg. Control (Y/N)	Content Neg. Control	Choice of Strains	Inoculation Concentration (cfu/g)	*Inoculation Technique* *(ID, AS, TS)*	Analysis Times (Day)
Dalzini et al., 2014 [15]	V. 2, 2008	GP	Turkey Bresaola	3	21 packs (100 g each)	Y	3 packs (100 g each)	Ref. strain ATCC^®^ 19115™ (DUP 1042) Field strain Lm171718 (DUP 1038) isolated from swine sausages Field strain Lm171767 (DUP 1038) isolated from swine sausages	32–100	AS	0, 40, 60, 90
Dalzini et al. 2014 [16]	V. 2, 2008	GP	Low-fat salami	3	42 trays (sliced)	Y	Not specified	Ref. strain ATCC^®^ 19115™ Collection strain Lm46113 Collection strain Lm168619	32–100	AS	0, 15, 30, 45, 60, 75, 90
Leong et al., 2015 [18]	V. 3, 2014	GP	Cold-smoked salmon	4 (2 each from facilities 1 and 2)	10 pieces of 30 or 50 g	N	N/A	Ref. strain 12MOB101LM Field strain 1123 (serotype 1/2a) isolated from smoked salmon Field strain 1319 (serotype 1/2a) isolated from smoked salmon	100	AS	0, afterwards every 2–3 days until approx. day 25
Novelli et al., 2017 [19]	V. 3, 2014	GP	Salami	12	Units of 0.8–1 kg	N	N/A	Field strain L1 isolated from salami produced locally Field strain L2 isolated from salami produced locally Ref. strain LMG13305	100	Contam. of dough (during production)	0, 7, 15, 25, 30, 40, 50 (overall summarized)
Hunt et al., 2018 [20]	V. 3, 2014	GP	Greek feta cheese Pork liver pâté Raw soft goat milk cheese Coleslaw Cold-smoked salmon	13 (2 batches/food type, 3 batches for goat’s milk cheese)	11 pieces of 25 g each, except for coleslaw	N	N/A	Field strain 6179 isolated from cheese processing environment Field strain 1020 isolated from raw bovine milk cheese Ref. strain 1382	100	AS	0, 7, 9, 14 (depending on food type)
Ruggeri et al., 2018 [17]	V. 2, 2008	GP	Salsiccia sarda Myrtle-flavored salsiccia sarda	3	15 samples/batch/tested ripening time	N	N/A	Ref. strain ATCC 35152 Wild-type strain serovar 1/2a recovered from salsiccia sarda samples Wild-type strain serovar 1/2c recovered from Salsiccia sarda samples	Approx. 10–100	Not specified	0, 225
Andritsos et al., 2019 [21]	V. 3, 2014	GP	Feta cheese-based Sauce	1	2 bulk packages (0.5kg each)	Y	Not specified	Ref. strain ATCC^®^ 19115™; serovar 4b Field strain AAL sample code 25060552 isolated from marinated veal	100	ID	0, 3, 5, 10, 15, 20, 25, 30, 35, 40
Marras et al., 2019 [22]	V. 3, 2014	GP	Ready-to-eat salad (radicchio 25%, endive 50%, chicory 25%)	3	60 pre-packaged mixes	Y	Not specified	Ref. strain ATCC 35152 2 wild-type strains isolated from the vegetable samples	Approx. 10–100	ID	0, 2, 4, 6, 8
Branciari et al., 2020 [23]	V. 3, 2014	GP, MGR	Soft Spreadable Salami	3	3 groups of 6 products (500 g each)	Y	1 group of 6 products (500 g each)	Ref. strain WDCM 00021 Field strain Lm15011/14 isolated from salami Field strain Lm36206/14 isolated from dry sausage	Approx. 100	Contam. of meat batter (during production)	0, 1, 2, 5, 10, 40, 70
Culliney et al., 2020 [27]	V. 3 Am. 1, 2019	GP, MGR	Iceberg lettuce Spinach Rocket	9 (3 batches/food type)	20 g samples	Y	8 control bags	Ref. strain 1382 Field strain 959 isolated from vegetables Field strain 6179 isolated from food processing plant	100	AS	0, 2, 5, 7, 9 (except iceberg lettuce)
Eicher et al., 2020 [24]	V. 3, 2014	GP	Norwegian smoked salmon Salmon fillet Sushi salmon	9 (3 batches/food type)	10 g samples	Y	1 × 10 g sample per salmon variety	Field strains: N18-1945, 5048, 121 (serotype 1/2a) N18-2495, 2812, 204, (serotype 1/2a) N18-2497, 1271, 5, (serotype 1/2b) (all from salmon processing food facility)	100	AS	Sushi salmon: 0, 2, 3 Norwegian smoked salmon: 0, 5, 12, 13, 14, 15, 16 Salmon fillets: 0, 5, 11, 12, 13, 14, 15
Collu et al., 2021 [25]	V. 3, 2014	GP	Coconut Fruit salad (grapes, kiwi, melon, pineapple) Pineapple Melon (piel de sapo) Melon (cantalupo)	3	6 packs each of coconut and melons, 15 packs each of fruit salad and pineapple, randomly selected from all 3 batches	Y	Not specified	Ref. strain ATCC 35152 Two wild-type strains isolated from ready-to-eat fruit samples	Approx. 10–100	ID	0, 2, 4, 6, 8, 10
Stella et al., 2021 [26]	V. 3, 2014	GP	Veal tartare	3	Portions 70 g each	Y	Blank samples	Ref. strains: 12MOB045LM 12MOB085LM 12MOB089LM	100	ID	0, 2, 5, 8, 10, 12
Vasileiadi et al., 2022 [28]	V. 4, 2021	GP	Soft Greek Anthotyros cheese	3	22 samples of 250 g each	Y	4 control units	Field strains: 09CEB411LM, serotype IIa, CC 26, ST 26, isolated from cheese 17SEL82LM, serotype IVb, CC 6, ST 6, isolated from cheese 17SEL22LM, serotype IIa, CC 14, ST 91, isolated from (environment) milk production filter	150	ID	0, 2, 7, 14, 23
Tirloni et al., 2023 [29]	V. 4, 2021	GP	Beef in tuna sauce	4	Sliced and packaged in 220 g portions	N	N/A	Ref. strains: 12MOB045LM 12MOB85LM 12MOB112LM	100	AS + ID	0, 4, 10, 15
Pniewski et al., 2024 [30]	V. 4, 2021	GP, MGR	Pork bars	3	50 g bars	Y	1 control group	Ref. strains: ATCC 7644TM ATCC 13932TM ATCC 1911TM	100	AS	0, 3, 7, 14, 21
Vasileiadi et al., 2024 [31]	V. 4, 2021	GP	Raw sea bass fillet	3	26 filet trays (90–110 g each)	Y	1 control unit, not further specified	Field strains: 03EB425LM, serotype IIa, CC 101, ST 775, isolated from fish 11CEB245LM, serotype IIa, CC 193, ST 193, isolated from fish 12MOB049LM, serotype IIb, CC 3, ST 3, isolated from the food industrial environment	50	AS	0, 4, 6, 8
Cipriani et al., 2025 [32]	V. 4, 2021	MGR	Tuna fillet Marinated salmon tartare Cubed salmon	9 (3/food type)	Tuna fillet: 1kg Marinated salmon tartare: 100 g Cubed salmon: 200 g	Y	Single product packs per food type	Field strain 12MOB099LM, serotype II, isolated from seafood	100	TS	0, 2, 3
Ștefan et al., 2025 [33]	V. 4, 2021	GP	Wiener sausage	6	7 samples (4 sticks of product per package)	Y	12 control samples for all batches	Ref. strain ATCC 13932 Field strain LM 1, isolated from meat products Field strain LM 2, isolated from meat products	Approx. 100	TS	0, 3, 5, 10, 15
Vasileiadi et al., 2025 [34]	V. 4, 2021	GP	Greek light cheese Greek full-fat semi-hard cheese	6 (3/food type)	20 × 200 g samples of ten slices	Y	4 samples/cheese type	Field strains: 17SEL22LM, serotype IIa, CC 14, ST 91, isolated from related environment 17SEL82LM, serotype IVb, CC 6, ST 6, isolated from raw milk cheese 09CEB411LM, serotype IIa, CC 26, ST 26, isolated from cheese	150	TS	0, 24, 59, 108, 165, 181

GP: growth potential; MGR: maximum growth rate; Y: yes; N: no; ID: in depth; AS: at surface; TS: through septum; N/A: not applicable; CC: clonal complex; ST: sequence type; LM: *Listeria monocytogenes*.

### Appendix A.2

**Table A2:** Summary of available data, continued.

Author	Storage Temperature	Results			
		Initial aw Value	Initial pH Value	End of Shelf Life aw Value	End of Shelf Life pH Value
Dalzini et al., 2014 [15]	5 °C for 7d, afterwards 8 °C for 83d	Average 0.923 ± 0.010 (range 0.911–0.939)	Average 5.55 ± 0.05 (range of 5.43–5.63)	Day 90: Average 0.925 ± 0.008 (range of 0.912–0.939)	Day 90: Average 5.45 ± 0.11 (range of 5.32–5.67)
Dalzini et al., 2014 [16]	8 °C for 7d, afterwards 12 °C for 83 days	Average 0.945 ± 0.005 (range 0.936–0.951)	Average 5.00 ± 0.10 (range 4.82–5.11)	Day 90: Average 0.939 ± 0.003 (range 0.935–0.949)	Day 90: No statistical difference (*p* > 0.05) for pH average
Leong et al., 2015 [18]	8 °C for 7d, afterwards 12 °C until approx. d25	Facility 1: 0.967 ± 0.001 Facility 2: 0.946 ± 0.023	Facility 1: 6.19 ± 0.08 Facility 2: 6.12 ± 0.05	Facility 1: 0.957 ± 0.001 Facility 2: 0.967 ± 0.000	Facility 1: 6.24 ± 0.04 Facility 2: 6.03 ± 0.01
Novelli et al., 2017 [19]	Conditions of each company	Not reported	Not reported	Not reported (decrease < 0.92 after 40 days)	Not reported (if GP > 0.5, internal pH: median 5.4, min. 5.0, max. 5.7 if GP < 0.5, internal pH: median 5.1, min. 4.6, max. 5.9)
Hunt et al., 2018 [20]	Smoked salmon: 6 °C for 5d and 8 °C for 9d Cheese and pork pâté: 8 °C for 14d Coleslaw: 8 °C for 7d and 12 °C for 14d	Coleslaw: 0.998 ± 0.001 Feta cheese: 0.973 ± 0.002 Goat milk cheese: 0.994 ± 0.002 Pork pate: 0.969 ± 0.004 Salmon 1: 0.979 ± 0.005 Salmon 2: 0.972 ± 0.004	Coleslaw: 5.49 ± 0.465 Feta cheese: 4.58 ± 0.155 Goat milk cheese: 4.32 ± 0.058 Pork pate: 6.20 ± 0.134 Salmon 1: 6.12 ± 0.065 Salmon 2: 6.26 ± 0.080	Day 14: Coleslaw: 0.985 ± 0.01 Feta cheese: 0.945 ± 0.018 Goat milk cheese: 0.986 ± 0.004 Pork pate: 0.964 ± 0.004 Salmon 1: 0.965 ± 0.002 Salmon 2: 0.950 ± 0.005	Day 14: Coleslaw: 6.51 ± 0.010 Feta cheese: 4.37 ± 0.150 Goat milk cheese: 4.15 ± 0.054 Pork pate: 5.89 ± 0.0320 Salmon 1: 6.11 ± 0.098 Salmon 2: 5.88 ± 0.110
Ruggeri et al., 2018 [17]	4 °C, 8 °C, 25 °C (for 12d and 20d of ripening each)	12 days ripening: 4 °C: 0.924 ± 0.007 8 °C: 0.924 ± 0.017 25 °C: 0.921 ± 0.005 20 days ripening: 4 °C: 0.881 ± 0.012 8 °C: 0.874 ± 0.010 25 °C: 0.868 ± 0.005	12 days ripening: 4 °C: 5.2 ± 0.22 8 °C: 5.4 ± 0.40 25 °C: 5.3 ± 0.12 20 days ripening: 4 °C: 5.7 ± 0.03 8 °C: 5.6 ± 0.35 25 °C: 5.77 ± 0.30	Day 225: 12 days ripening: 4 °C: 0.912 ± 0.010 8 °C: 0.901 ± 0.010 25 °C: 0.881 ± 0.004 20 days ripening: 4 °C: 0.852 ± 0.012 8 °C: 0.862 ± 0.007 25 °C: 0.861 ± 0.007	Day 225: 12 days ripening: 4 °C: 5.8 ± 0.27 8 °C: 5.7 ± 0.32 25 °C: 5.8 ± 0.25 20 days ripening: 4 °C: 5.8 ± 0.27 8 °C: 5.8 ± 0.02 25 °C: 5.9 ± 0.27
Andritsos et al., 2019 [21]	4 °C for 30 days (4.3 ± 0.4 °C)	Not reported	4.6	Not reported	4.0
Marras et al., 2019 [22]	4 °C, 8 °C, 25 °C, 37 °C	4 °C: 0.996 ± 0.003 8 °C: 0.992 ± 0.002 25 °C: 0.991 ± 0.002 37 °C: 0.998 ± 0.004	4 °C: 6.35 ± 0.057 8 °C: 6.23 ± 0.025 25 °C: 6.19 ± 0.080 37 °C: 6.15 ± 0.076	Day 8: 4 °C: 0.991 ± 0.003 8 °C: 0.985 ± 0.006 25 °C: 0.986 ± 0.006 37 °C: 0.994 ± 0.002	Day 8: 4 °C: 6.47 ± 0.014 8 °C: 6.97 ± 0.14 25 °C: 6.03 ± 0.14 37 °C: 5.44 ± 0.38
Branciari et al., 2020 [23]	8 °C for 7d and 12 °C for 53d	Mean 0.961	Mean 5.82	Day 70: Mean 0.931	Day 70: Mean 5.44
Culliney et al., 2020 [27]	8 °C ± 0.5 °C for 9d	Spinach: 0.974 ± 0.001 Rocket: 0.980 ± 0.002 Lettuce: 0.993 ± 0.001	Spinach: 7.30 ± 0.0078 Rocket: 6.55 ± 0.153 Lettuce: 6.34 ± 0.029	Day 9: Spinach: 0.970 ± 0.002 Rocket: 0.976 ± 0.001 Day 7, Lettuce: 0.991 ± 0.005	Day 9: Spinach: 7.25 ± 0.0038 Rocket: 6.86 ± 0.086 Day 7, Lettuce: 6.36 ± 0.142
Eicher et al., 2020 [24]	5 °C and 8 °C throughout the shelf life	Norwegian smoked salmon: 0.96 ± 0.002 Salmon filet: 0.97 ± 0.006 Sushi salmon: 0.99 ± 0.003	Norwegian smoked salmon: 6.0 ± 0.0 Salmon filet: 6.0 ± 0.1 Sushi salmon: 6.1 ± 0.1	Norwegian smoked salmon: 0.96 ± 0.002 (overall) Salmon filet: 0.97 ± 0.006 (overall) Sushi salmon: 0.99 ± 0.003 (overall)	Norwegian smoked salmon: 6.0 ± 0.0 (overall) Salmon filet: 6.0 ± 0.1 (overall) Sushi salmon: 6.1 ± 0.1 (overall)
Collu et al., 2021 [25]	4 °C and 8 °C for 10d each	Not reported	Not reported	(all averages and SD) Pineapple: 0.989 ± 0.0001 Melon (both): 0.991 ± 0.0004 Fruit salad: 0.998 ± 0.0003 Coconut: 0.990 ± 0.0008	(all averages and SD) Pineapple: 3.6 ± 0.015 Melon (both): 5.58 ± 0.018 Fruit salad: 3.74 ± 0.015 Coconut: 6.27 ± 0.006
Stella et al., 2021 [26]	8 °C for 12d	0.98–0.99 (all batches, all sampling times)	5.36–5.54 (all batches)	0.98–0.99 (all batches, all sampling times)	Day 12: 4.94–5.10 (all batches)
Vasileiadi et al., 2022 [28]	5 °C for 2 days, 7 °C for 12 days, 10 °C for 9 days	All control units, all averages and SD: Batch 1: 0.96 ± 0.94 Batch 2: 0.97 ± 0.01 Batch 3: 0.97 ± 0.01	All control units, all averages and SD: Batch 1: 6.50 ± 0.04 Batch 2: 6.80 ± 0.07 Batch 3: 6.66 ± 0.09	Day 23, all control units, all averages and SD: Batch 1: 0.95 ± 0.00 Batch 2: 0.96 ± 0.01 Batch 3: 0.96 ± 0.01	Day 23, all control units, all averages and SD: Batch 1: 6.33 ± 0.16 Batch 2: 6.40 ± 0.13 Batch 3: 6.32 ± 0.04
Tirloni et al., 2023 [29]	8 °C for 15 days	Not reported	Not reported	Not reported	Not reported
Pniewski et al., 2024 [30]	2 °C, 4 °C, 6 °C for 21 days	(control bars) 2 °C: 0.952 ± 0.001 4 °C: 0.952 ± 0.001 6 °C: 0.952 ± 0.001	(control bars) 2 °C: 6.06 ± 0.02 4 °C: 6.06 ± 0.02 6 °C: 6.06 ± 0.02	Day 21 (control bars): 2 °C: 0.949 ± 0.001 4 °C: 0.946 ± 0.002 6 °C: 0.943 ± 0.002	Day 21 (control bars): 2 °C: 6.02 ± 0.01 4 °C: 6.08 ± 0.01 6 °C: 6.08 ± 0.02
Vasileiadi et al., 2024 [31]	2 °C for 2 days, 4 °C for 4 days, 10 °C for 2 days	Control units (average and SD): Batch 4: 0.99 ± 0.01 Batch 5: 0.989 ± 0.001 Batch 6: 0.990 ± 0.004	Control units (average and SD): Batch 4: 6.555 ± 0.106 Batch 5: 6.495 ± 0.035 Batch 6: 6.43 ± 0.099	Control units (average and SD): Batch 4: 0.99 ± 0.01 Batch 5: 0.989 ± 0.001 Batch 6: 0.990 ± 0.004	Control units (average and SD): Batch 4: 6.555 ± 0.106 Batch 5: 6.495 ± 0.035 Batch 6: 6.43 ± 0.099
Cipriani et al., 2025 [32]	10 °C for 9 days or 7 days	(all means and SD, all food control units) Tuna fillet: 0.99 ± 0.01 Marinated salmon tartare: 0.97 ± 0.01 Cubed salmon: 0.99 ± 0.01	(all means and SD, all food control units) Tuna fillet: 5.9 ± 0.1 Marinated salmon tartare: 5.9 ± 0.1 Cubed salmon: 6.2 ± 0.1	(all means and SD, all food control units) Tuna fillet: 0.98 ± 0.01 Marinated salmon tartare: 0.96 ± 0.01 Cubed salmon: 0.99 ± 0.01	(all means and SD, all food control units) Tuna fillet: 5.9 ± 0.1 Marinated salmon tartare: 5.9 ± 0.1 Cubed salmon: 6.1 ± 0.3
Ștefan et al., 2025 [33]	7 °C for 15 days	(all means and SD) Batch 1: 0.952 ± 0.001 Batch 2: 0.947 ± 0.001 Batch 3: 0.948 ± 0.002 Batch 4: 0.950 ± 0.001 Batch 5: 0.945 ± 0.003 Batch 6: 0.946 ± 0.0002	(all means and SD) Batch 1: 5.76 ± 0.45 Batch 2: 5.66 ± 0.40 Batch 3: 5.74 ± 0.35 Batch 4: 6.15 ± 0.30 Batch 5: 6.18 ± 0.35 Batch 6: 5.89 ± 0.40	Day 15 (all means and SD): Batch 1: 0.945 ± 0.002 Batch 2: 0.940 ± 0.002 Batch 3: 0.943 ± 0.001 Batch 4: 0.946 ± 0.001 Batch 5: 0.943 ± 0.001 Batch 6: 0.942 ± 0.001	Day 15 (all means and SD): Batch 1: 5.89 ± 0.35 Batch 2: 5.82 ± 0.40 Batch 3: 5.90 ± 0.35 Batch 4: 6.32 ± 0.45 Batch 5: 6.30 ± 0.40 Batch 6: 6.27 ± 0.35
Vasileiadi et al., 2025 [34]	5 °C for 24 days, 7 °C for 35 days, 7 °C for 49 days, 7 °C for 57 days, 10 °C for 17 days	Light semi-hard sliced cheese (all control samples, all averages and SD): Batch 1: 0.78 ± 0.03 Batch 2: 0.72 ± 0.03 Batch 3: 0.70 ± 0.03 Full-fat semi-hard sliced cheese (all control samples, all averages and SD): Batch 1: 0.74 ± 0.06 Batch 2: 0.776 ± 0.008 Batch 3: 0.784 ± 0.004	Light semi-hard sliced cheese (all control samples, all averages and SD): Batch 1: 5.84 ± 0.21 Batch 2: 6.21 ± 0.13 Batch 3: 5.76 ± 0.08 Full-fat semi-hard sliced cheese (all control samples, all averages and SD): Batch 1: 5.99 ± 0.21 Batch 2: 5.70 ± 0.11 Batch 3: 6.00 ± 0.11	Day 181, Light semi-hard sliced cheese (all control units, all averages and SD): Batch 1: 0.69 ± 0.05 Batch 2: 0.74 ± 0.04 Batch 3: 0.75 ± 0.04 Day 181, Full-fat semi-hard sliced cheese (all control samples, all averages and SD): Batch 1: 0.73 ± 0.03 Batch 2: 0.749 ± 0.001 Batch 3: 0.798 ± 0.017	Day 181, Light semi-hard sliced cheese (all control units, all averages and SD): Batch 1: 6.33 ± 0.13 Batch 2: 6.45 ± 0.07 Batch 3: 6.40 ± 0.26 Day 181, Full-fat semi-hard sliced cheese (all control samples, all averages and SD): Batch 1: 6.18 ± 0.05 Batch 2: 5.92 ± 0.01 Batch 3: 6.47 ± 0.57

SD: standard deviation.

### Appendix A.3

**Table A3:** Summary of available data, continued.

Author	Results					
	Contamination Control Samples (N, P)	Initial Count *L. monocytogenes* (log cfu/g)	End of Shelf Life Count *L. monocytogenes* (log cfu/g)	GP (δ)	Growth > 0.5 log cfu/g (Yes/No)	MGR (u max)
Dalzini et al., 2014 [15]	N	(all averages and SD) Batch 1: 1.68 ± 0.28 Batch 2: 1.81 ± 0.17 Batch 3: 1.50 ± 0.13	Day 90: Batch 1: 1.03 ± 0.07 Batch 2 and 3: not detectable (<0.47)	Range: −1.32 (batch 2) to −0.58 (batch 1)	No	N/A
Dalzini et al., 2014 [16]	N	(all means and SD) Batch 1: 1.79 ± 0.03 Batch 2: 1.44 ± 0.22 Batch 3: 1.64 ± 0.13	Not detectable in any batch	Batch 1: −1.30 Batch 2: −0.91 Batch 3: −1.18	No	N/A
Leong et al., 2015 [18]	N/A	Facility 1, Batch 1: 1.87 ± 0.23 Facility 1, Batch 2: 1.75 ± 0.36 Facility 2, Batch 1: 2.15 ± 0.16 Facility 2, Batch 2: 2.23 ± 0.30	Facility 1: significant increase in both batches, followed by decrease over the course of challenge test Facility 2: significant increase in both batches over the course of challenge test	Facility 1, Batch 1: 2.57 Facility 1, Batch 2: 5.15 Facility 2, Batch 1: 2.94 Facility 2, Batch 2: 1.63	Yes	N/A
Novelli et al., 2017 [19]	N/A	Not reported	Not reported	4/12 challenge tests: >0.5 8/12 challenge tests: <0.5	4/12 challenge tests: Yes 8/12 challenge tests: No	N/A
Hunt et al., 2018 [20]	N/A	Coleslaw Batch 1: 2.62, 2.30, 2.45 Coleslaw Batch 2: 2.54, 2.50, 2.51 Feta cheese Batch 1: 2.14, 2.26, 2.38, 2.08 Feta cheese Batch 2: 2.60, 2.56, 2.64, 2.34 Goat milk cheese Batch 1: 1.78, 1.90 Goat milk cheese Batch 2: 1.90, 1.90 Goat milk cheese Batch 3: 1.78, 1.48 Pork pate Batch 1: 1.75, 1,81 Pork pate Batch 2: 1.90, 1.90 Smoked salmon A Batch 1: 1.83, 1.68 Smoked salmon A Batch 2: 1.60, 1.51 Smoked salmon B Batch 1: 1.90, 1.78 Smoked salmon B Batch 2: 1.90, 2.00	Day 14, cheeses and coleslaw: <1 Day 7, pork pate: Not determined as growth observed Day 9, smoked salmon A Batch 1: 4.92, 5.19 Day 9, smoked salmon A Batch 2: 4.59, 3.92 Day 9, smoked salmon B Batch 1: 3.81, 3.64 Day 9, smoked salmon B Batch 2: 3.60, 3.58	Cheeses and coleslaw: <0.5 Pork pate and smoked salmon: >0.5 in at least 1 replicate	Cheeses and coleslaw: No Pork pate and smoked salmon: Yes (in at least 1 replicate)	N/A
Ruggeri et al., 2018 [17] *	N/A	12 days ripening: 4 °C: 1.66 8 °C: 1.6 25 °C: 1.4 20 days ripening: 4 °C: 1.48 8 °C: 1.48 25 °C: 1.3	Day 225: 12 days ripening: 4 °C, 8 °C, 25 °C: <1 (median) Day 225: 20 days ripening: 4 °C, 8 °C, 25 °C: <1 (median)	12 days ripening: 4 °C: −1.35 8 °C: −1.6 25 °C: −0.70 20 days ripening: 4 °C: −1.17 8 °C: −1.00 25 °C: −0.99	No	N/A
Andritsos et al., 2019 [21]	N	Not reported	Day 30 (end of shelf life: d40): 1.1 (mean contamination level overall: 2.3)	−1.2	No	N/A
Marras et al., 2019 [22]	N	(all medians) 4 °C, 8 °C, 25 °C, 37 °C: 1.40	Day 8 (all medians): 4 °C: 2.04 8 °C: 2.85 25 °C: 7.34 37 °C: 7.72	4 °C: 0.044 8 °C: 1.45 25 °C: 0.274 37 °C: 6.32	4 °C, 25 °C: No 8 °C, 37 °C: Yes	N/A
Branciari et al., 2020 [23] *	N	Ripening period (all medians and SD): Batch 1: 2.15 ± 0.02 Batch 2: 2.23 ± 0.09 Batch 3: 2.10 ± 0.04 Storage period (all medians and SD) Batch 1: 2.28 ± 0.14 Batch 2: 2.63 ± 0.48 Batch 3: 2.50 ± 0.08	Ripening period, Day 10 (all medians and SD): Batch 1: 2.28 ± 0.14 Batch 2: 2.63 ± 0.48 Batch 3: 2.50 ± 0.08 Storage period, Day 70 (all medians and SD) Batch 1: <1 ± 0 Batch 2: 1 ± 0.28 Batch 3: 1.10 ± 0.09	Ripening period: Batch 1: 0.13 Batch 2: 0.40 Batch 3: 0.40 Storage period: Batch 1: >−1.28 Batch 2: −1.63 Batch 3: −1.28	No	(all in log cfu/h) Batch 1: −0.0019 Batch 2: −0.0013 Batch 3: −0.0014
Culliney et al., 2020 [27]	N	(all medians) Spinach: Batch 1: 1.98 Batch 2: 1.73 Batch 3: 2.02 Rocket: Batch 1: 2.00 Batch 2: 1.61 Batch 3: 1.99 Lettuce: Batch 1: 1.79 Batch 2: 1.96 Batch 3: 1.34	(all medians) Day 9, Spinach: Batch 1: 4.34 Batch 2: 4.51 Batch 3: 4.85 Day 9, Rocket: Batch 1: 3.67 Batch 2: 3.55 Batch 3: 3.86 Day 7, Lettuce: Batch 1: 3.07 Batch 2: 3.65 Batch 3: 3.96	Spinach: Batch 1: 2.36 Batch 2: 2.78 Batch 3: 2.83 Rocket: Batch 1: 1.67 Batch 2: 1.94 Batch 3: 1.87 Lettuce: Batch 1: 1.28 Batch 2: 1.69 Batch 3: 2.62	Yes	(all in log cfu day^−1^) Spinach: Batch 1: 0.266 Batch 2: 0.326 Batch 3: 0.336 Rocket: Batch 1: 0.210 Batch 2: 0.225 Batch 3: 0.184 Lettuce: Batch 1: 0.193 Batch 2: 0.377 Batch 3: 0.235
Eicher et al., 2020 [24]	Not reported	(all means) Salmon fillet, 5 °C: 1.9 Salmon fillet, 8 °C: 1.9 Sushi salmon, 5 °C: 1.8 Sushi salmon, 8 °C: 1.8 Norwegian smoked salmon, low NaL, 5 °C: 1.7 Norwegian smoked salmon, low NaL, 8 °C: 1.7 Norwegian smoked salmon, high NaL, 5 °C: 2.7 Norwegian smoked salmon, high NaL, 8 °C; mean 2.7	(all means and ± SD) Day 15, 5 °C, Salmon fillet: 4.6 ± 1.14 Day 15, 8 °C, Salmon fillet: 5.5 ± 0.75 Day 3, 5 °C, Sushi salmon: 2.8 Day 3, 8 °C, Sushi salmon: 3.5 Day 16, 5 °C, Norwegian smoked salmon, low NaL: 3.4 Day 16, 8 °C, Norwegian smoked salmon, low NaL: 5.2	Sushi salmon, 5 °C: Replicate 1: 0.87 Replicate 2: 0.95 Replicate 3: 1.05 Sushi salmon, 8 °C: Replicate 1: 1.16 Replicate 2: 1.87 Replicate 3: 1.98 Norwegian smoked salmon (low NaL), 5 °C: Replicate 1: 2.21 Replicate 2: 1.82 Replicate 3: 0.69 Norwegian smoked salmon (low NaL), 8 °C: Replicate 1: 3.57 Replicate 2: 3.47 Replicate 3: 2.87 Norwegian smoked salmon (high NaL), 5 °C: Replicate 1: −0.09 Replicate 2: 0.24 Replicate 3: 0.32 Norwegian smoked salmon (high NaL), 8 °C: Replicate 1: 1.19 Replicate 2: 2.12 Replicate 3: 2.30 Salmon fillet, 5 °C: Replicate 1: 2.78 Replicate 2: 3.78 Replicate 3: 1.30 Salmon fillet, 8 °C: Replicate 1: 2.83 Replicate 2: 4.70 Replicate 3: 3.33	Norwegian smoked salmon (high NaL), 5 °C: No (all replicates) Rest: Yes	N/A
Collu et al., 2021 [25]	Not reported	Not reported	Not reported	4 °C: Pineapple: −0.52 Fruit salad: −0.59 Melon (cantaloupe): 0.13 Melon (piel de sapo): 0.58 8 °C: Pineapple: −0.55 Fruit salad: −0.69 Melon (cantaloupe): 0.97 Melon (piel de sapo): 1.50	4 °C, Melon (piel de sapo): Yes 4 °C, Rest: No 8 °C, Pineapple, Fruit salad: No 8 °C, Melon (cantaloupe), Melon (piel de sapo): Yes	N/A
Stella et al., 2021 [26]	N	Batch 1: 1.40, 1.60, 1.88 Batch 2: 1.74, 1.85, 1.88 Batch 3: 1.74, 1.78, 1.88	Batch 1: 1.30, 1.78, 1.85 Batch 2: 0.60, 0.70, 1.00 Batch 3: 1.48, 1.48, 1.78	Batch 1: 0.18 Batch 2: −1.15 Batch 3: −0.30	No	N/A
Vasileiadi et al., 2022 [28]	N (not detected in 25 g)	Batch 1: 3.10 Batch 2: 2.93 Batch 3: 2.23	Day 23: Batch 1: 6.36 Batch 2: 6.71 Batch 3: 7.04	Batch 1: 3.33 Batch 2: 4.14 Batch 3: 4.94	Yes	N/A
Tirloni et al., 2023 [29]	Not reported	(All means and SD) Upper beef slice: 2.93 ± 0.10 In sauce: 2.96 ± 0.12 Between beef slices: 2.62 ± 0.54	Day 15 (all means and SD): Upper beef slice: 3.36 ± 0.29 In sauce: 3.03 ± 0.11 Between beef slices: 2.77 ± 0.42	Upper beef slice: 0.71 In sauce: 0.25 Between beef slices: 0.60	In sauce: No Rest: Yes	N/A
Pniewski et al., 2024 [30]	N (no detection of any viable *L. monocytogenes* cells using ISO 11290-2:2017 method, confirmed by ELFA technique, corresponding to absence in 25 g)	2 °C: Batch 1: 2.05 ± 0.06 Batch 2: 2.12 ± 0.04 Batch 3: 2.10 ± 0.12 4 °C: Batch 1: 2.05 ± 0.06 Batch 2: 2.12 ± 0.04 Batch 3: 2.10 ± 0.12 6 °C: Batch 1: 2.05 ± 0.07 Batch 2: 2.12 ± 0.05 Batch 3: 2.10 ± 0.13	Day 21, 2 °C: Batch 1: 0.60 ± 0.22 Batch 2: 0.95 ± 0.09 Batch 3: 0.88 ± 0.14 Day 21, 4 °C: Batch 1: 0.95 ± 0.25 Batch 2: 1.34 ± 0.11 Batch 3: 1.26 ± 0.15 Day 21, 6 °C: Batch 1: 2.96 ± 0.16 Batch 2: 2.72 ± 0.10 Batch 3: 2.68 ± 0.10	2 °C: 0.36 4 °C: 0.14 6 °C: 0.91	2 °C, 4 °C: No 6 °C: Yes	(Measurement unit not reported) 2 °C and 4 °C: negative MGR 6 °C: 0.0493 ± 0.0303
Vasileiadi et al., 2024 [31]	N	Batch 4: 1.70, 1.78, 1.78 Batch 5: 1.74, 1.85, 1.90 Batch 6: 1.60, 1.78, 1.88	Day 8: Batch 4: 4.16 Batch 5: 4.46 Batch 6: 4.11	Batch 4: 2.41 Batch 5: 2.63 Batch 6: 2.36	Yes	N/A
Cipriani et al., 2025 [32]	Not reported	Tuna filet: Batch 1: 1.7 ± 0.2 Batch 2: 2.1 ± 0.4 Batch 3: 1.5 ± 0.4 Marinated salmon tartare: Batch 1: 2.6 ± 0.2 Batch 2: 2.6 ± 0.1 Batch 3: 2.1 ± 0.1 Cubed salmon: Batch 1: 2.2 ± 0.1 Batch 2: 2.8 ± 0.1 Batch 3: 2.0 ± 0.1	Tuna filet: Batch 1: 5.9 ± 0.1 Batch 2: 6.8 ± 0.3 Batch 3: 6.9 ± 0.5 Marinated salmon tartare: Batch 1: 5.5 ± 0.4 Batches 2 and 3: not available Cubed salmon: Batch 1: 4.3 ± 0.1 Batch 2: 6.6 ± 0.1 Batch 3: 4.7 ± 0.1	N/A	N/A	(all in log cfu/h) Tuna filet: Batch 1: 0.049 ± 0.009 Batch 2: 0.038 ± 0.006 Batch 3: 0.035 ± 0.006 Marinated salmon tartare: Batch 1: 0.019 ± 0.003 Batch 2: 0.020 ± 0.002 Batch 3: 0.022 ± 0.002 Cubed salmon: Batch 1: 0.033 ± 0.001 Batch 2: 0.042 ± 0.002 Batch 3: 0.042 ± 0.002
Ștefan et al., 2025 [33]	N (absent in 25 g)	Batch 1: 2.66, 2.76, 2.72 Batch 2: 2.63, 2.67, 2.68 Batch 3: 2.59, 2.72, 2.79 Batch 4: 2.79, 2.84, 2.70 Batch 5: 2.83, 2.87, 2.86 Batch 6: 2.88, 2.93, 2.92	Day 15 (all means): Batch 1: 3.21 Batch 2: 3.14 Batch 3: 3.35 Batch 4: 5.72 Batch 5: 5.76 Batch 6: 5.81	Batch 1: 0.77 Batch 2: 0.71 Batch 3: 0.82 Batch 4: 2.94 Batch 5: 2.91 Batch 6: 2.90	Yes	N/A
Vasileiadi et al., 2025 [34]	N (not detected in 25 g)	Light semi-hard sliced cheese: Batch 1: 2.08 Batch 2: 2.31 Batch 3: 2.23 Full-fat semi-hard sliced cheese: Batch 1: 2.20 Batch 2: 2.02 Batch 3: 2.08	Day 181, Light semi-hard sliced cheese: Batch 1: <1.60 Batch 2: <1.60 Batch 3: <1.60 Day 181, Full-fat semi-hard sliced cheese: Batch 1: <1.00 Batch 2: <1.60 Batch 3: <1.00	Not calculable due to the downward trend	N/A	N/A

GP: growth potential; MGR: maximum growth rate; N/A: not applicable; N: negative; P: positive; SD: standard deviation. * At the time the referenced study was conducted (2018), ISO 20976-2:2022 was not yet available. Therefore, a growth potential challenge test was applied. According to current guidance, studies aiming to evaluate ripening effects would more specifically address inactivation kinetics.
